# Modeling pressure drop values across ultra-thin nanofiber filters with various ranges of filtration parameters under an aerodynamic slip effect

**DOI:** 10.1038/s41598-023-32765-4

**Published:** 2023-04-03

**Authors:** Songhui Lee, Dai Bui-Vinh, Minwoo Baek, Dong-Bin Kwak, Handol Lee

**Affiliations:** 1 Department of Environmental Engineering, Inha University, 100 Inha-ro, Michuhol-gu, Incheon, 22212 Republic of Korea; 2 Particle Technology Laboratory, Mechanical Engineering, University of Minnesota, 111 Church St., S.E., Minneapolis, 55455 USA

**Keywords:** Fluid dynamics, Environmental sciences, Engineering

## Abstract

Computational fluid dynamics simulations of fibrous filters with 56 combinations of different fiber sizes, packing densities, face velocities, and thicknesses were conducted for developing models that predict pressure drops across nanofiber filters. The accuracy of the simulation method was confirmed by comparing the numerical pressure drops to the experimental data obtained for polyacrylonitrile electrospun nanofiber filters. In the simulations, an aerodynamic slip effect around the surface of the small nanofibers was considered. The results showed that, unlike in the case of conventional filtration theory, pressure drops across the thin layers of electrospun nanofiber filters are not proportional to the thickness. This might be a critical factor for obtaining precise pressure drops across the electrospun nanofiber filters with extremely thin layers. Finally, we derived the product of drag coefficient and Reynolds number as a function of packing density, Knudsen number, and ratio of thickness to fiber diameter to get the correlation equation for pressure drop prediction. The obtained equation predicted the pressure drops across the nanofiber filters with the maximum relative difference of less than 15%.

## Introduction

The negative impact of air pollution cannot be overemphasized. It threatens the human respiratory system and thus causes severe health issues including heart disease, pneumonia, stroke, diabetes, and lung cancer^1–4^. Because millions of deaths annually are estimated to be caused by exposure to indoor and outdoor air pollution, the World Health Organization (WHO) considers air pollution to be the single largest environmental health risk^5,6^. Particulate matter (PM), a complex mixture of fine solid and liquid particles with various chemical compositions, is one of the primary air pollutants^1,7^. The small size and large surface area of PM_2.5_ (aerodynamic size less than 2.5 µm) can penetrate deep into the human lungs and can be toxic, thus increasing morbidity and mortality^8–11^. Therefore, it is of significant importance to effectively control and remove PM from daily lives of humans.

Air filtration is considered one of the most effective methods to control air quality. This is generally achieved using membrane and fibrous materials^12,13^. The performance of filter materials can be assessed using diverse metrics^14^. In general, the performance of filters is evaluated by quality factor (${\text{QF}} = - \ln \;(1 - \eta )/\Delta P$); therefore, the pressure drop (Δ*P*) across filter media is an important factor in addition to the removal efficiency (*η*) measured in terms of energy consumption^15,16^. Fibrous type air filters are widely employed in many filtration applications due to their highly porous structures (i.e., low packing density) compared to membrane type filters^17^. Conventional fibrous air filters consist of fibers with diverse diameters from a few microns to tens of microns. These large-sized fibers require a substantial thickness to capture PM with a high removal efficiency, which induces large pressure drops. To address this trade-off between efficiency and pressure drop, nanofiber filters with the fiber sizes from tens of nanometers to hundreds of nanometers, which are produced by electrospinning process, have attracted significant attention^18–22^. One of the distinct advantages of nanofiber filters is that due to the aerodynamic slip around the surface of the small nanofibers, resistance against gas flow is reduced, which leads to lower pressure drops across individual fibers^23–26^.

To develop this promising technique, many researchers have performed experimental investigations related to performance metrics such as mechanical strength, removal efficiency, and pressure drop^27^. Leung et al.^28^ examined the effects of packing density and thickness on removal efficiency and pressure drop by stacking layers of polyethylene oxide nanofibers with 208 nm mean diameter. Their study revealed that the multi-layering nanofiber filters considerably reduce the pressure drop compared to the single layer with the same amount of nanofiber deposition. Zhang et al.^29^ developed electrospun polyimide nanofiber filters with high-temperature stability for the application of PM_2.5_ removal from car exhaust gas. Xia et al.^30^ examined the relationship between the pressure drop and face velocity for electrospun nanofibers by collecting 122 experimental data from the literature. In addition, computational fluid dynamics (CFD) simulations have been used to investigate the complex flow characteristics inside nanofiber filters as the numerical approach has the advantage of simplified adjustment of the filtration parameters^31^. Hosseini^32^ developed 3-D CFD simulations for estimating pressure drops at different packing densities and fiber sizes. Quan et al.^33^ numerically modeled the slip effect on a single nanofiber to find the optimal slip effect functional fibers, which could be applicable to sandwich structured fibrous filters for reducing pressure drops.

Several studies investigated the prediction of pressure drops across fibrous filters. A theoretical model for predicting pressure drops in Kuwabara^34^ by assuming that the same-sized fibers are evenly distributed perpendicular to the flow. Davies^35^ proposed an empirical expression of pressure drop across fibers valid for the packing density less than 0.3. However, the pressure drop equations in Kuwabara^34^ and Davies^35^ did not consider the slip effect that reduces the hydrodynamic drag on nanofibers. Brown^36^ suggested a theoretical equation modified from Kuwabara’s model with the slip effect for nanofibers. Recently, an empirical equation for predicting pressure drop across nanofiber was provided by Bian et al.^37^ based on the experimental pressure drops from 25 nylon electrospun nanofibers.

Despite the above-mentioned studies on the performance of electrospun nanofibers, the previous research has focused mainly on improving the removal efficiency and pressure drop, developing simulation tools, or examination of the effects of the limited filtration parameters. Therefore, combined effects of the important parameters such as fiber size, packing density, face velocity, and thickness on the filtration performance should be further studied. In this study, CFD simulations of 56 cases with various conditions of fiber size (50–800 nm), packing density (0.02–0.08), face velocity (5–20 cm/s), and thickness (0.25–80 µm), which are the dominant parameters of typical electrospun nanofiber filters, were conducted using randomly placed nanofibers. Based on the conventional filtration theory and the numerically obtained pressure drops, correlation equation as a function of the parameters were developed to predict pressure drops across ultra-thin nanofiber filters.

## Method

### Theoretical approach

Based on the drag theory, which suggests that the pressure drop of clean fibrous filters depends on the force balance, Wong^38^ proposed the pressure drop across fibrous media, Δ*P*, as follows:

1 $$ \Delta P = C_{D} \frac{{2\rho \alpha U_{0}^{2} L}}{{\pi d_{f} }}, $$

where, *C*_*D*_ is the drag coefficient, *ρ* is the fluid density, *α* is the fiber packing density, *L* is the filter thickness, *d*_*f*_ is the fiber diameter, and *U*_*0*_ is the face velocity. Moreover, White^39^ found that the drag coefficient of fibrous filters is inversely proportional to Reynolds number (${\text{Re}} = \frac{{\rho U_{0} d_{f} }}{\mu }$) and can be correlated with the packing density, i.e., $\frac{{C_{D} }}{2} \cdot {\text{Re}} = f\;(\alpha )$. Therefore, Eq. (1) can be rearranged as follows:

2 $$ \Delta P = \frac{4}{\pi }\left( {\frac{{C_{D} }}{2} \cdot {\text{Re}}} \right)\frac{{\mu \alpha U_{0} L}}{{d_{f}^{2} }}, $$

where, *μ* is the fluid dynamic viscosity. However, a considerable aerodynamic slip occurs when the fiber diameter is comparable to the mean free path (*λ*) of gas molecules, e.g., around 67 nm for air at standard pressure and temperature. Kirsch^24^ reported that when a significant slip occurs, Knudsen number (${\text{Kn}} = \frac{2\lambda }{{d_{f} }}$) should be considered in the drag force term, i.e., $\frac{{C_{D} }}{2} \cdot {\text{Re}} = f\;(\alpha ,\;Kn)$. Furthermore, the typical layer thickness of electrospun nanofibers is considerably thin, ranging from a few hundred nanometers to tens of micrometers. In these extremely thin nanofiber layers, the arrangement of nanofibers can significantly influence the drag force^30,40^, which might be increasingly pronounced in the case of thinner layers, and we assumed that the effect of the fiber arrangement is related to the ratio of thickness to fiber diameter, $\frac{L}{{d_{f} }}$, by adding this non-dimensional form of thickness as a variable to the drag force term. Finally, the pressure drop across the electrospun nanofiber filters, that is, Eq. (2), can be expressed as:

3 $$ \Delta P = \frac{4}{\pi } \cdot f\left( {\alpha ,\;{\text{Kn}},\;\frac{L}{{d_{f} }}} \right)\frac{{\mu \alpha U_{0} L}}{{d_{f}^{2} }}. $$

### Numerical approach

#### Simulation conditions

In this study, we suggested the model for predicting the pressure drop of electrospun nanofiber filters by obtaining $\frac{{C_{D} }}{2} \cdot {\text{Re}}$ as a function of packing density, Kn, and the ratio of thickness to fiber diameter using 56 cases of different filtration parameter combinations considering typical electrospun nanofiber filters. As shown in Table 1, we considered fiber diameters from 50 to 800 nm, corresponding to Kn from 2.669 to 0.167, respectively. The packing densities of the fiber filters from 0.02 to 0.08 were examined based on the generally observed range of packing densities of electrospun nanofiber filters. The face velocities of 5, 10, 15, and 20 cm/s were used. Typically, electrospun nanofiber filters are employed in the low velocity range because of their weak mechanical strength, which is attributed to their low packing density and small fiber size relative to conventional filters, e.g., non-woven filters. The filter thickness ranges from 0.25 to 80 µm, that is, from ultra-thin to relatively thick layers.

**Table 1:** Filtration parameters of 56 cases for the numerical study on nanofiber filters.

*d*_*f*_ (nm)	*α* (–)	*U*_*0*_ (cm/s)	*L* (μm)	Kn	Effect
50	0.02, 0.04, 0.06, 0.08	5	10	2.669	Packing density
100	0.02, 0.04, 0.06, 0.08	5	20	1.334	
200	0.02, 0.04, 0.06, 0.08	5	40	0.667	
400	0.02, 0.04, 0.06, 0.08	5	80	0.334	
800	0.02, 0.04, 0.06, 0.08	5	80	0.167	
100	0.04	5	10, 40, 80	1.334	Thickness
200	0.04	10, 15, 20	40	0.667	Face velocity
50, 200, 400, 800	0.04	5	20	2.669, 0.667, 0.334, 0.167	Fiber size (or Kn)
50	0.06	5	0.25, 0.5, 1, 2, 4	2.669	Thickness
100	0.06	5	0.5, 1, 2, 4	1.334	
200	0.06	5	1, 2, 4	0.667	
400	0.06	5	2, 4	0.334	
800	0.06	5	2, 4	0.167	
50	0.04, 0.08	5	0.25	2.669	Packing density
100	0.04, 0.08	5	0.5	1.334	
200	0.04, 0.08	5	1	0.667	
400	0.04, 0.08	5	2	0.334	
800	0.04, 0.08	5	4	0.167	

#### Simulation domain

**Figure 1:**
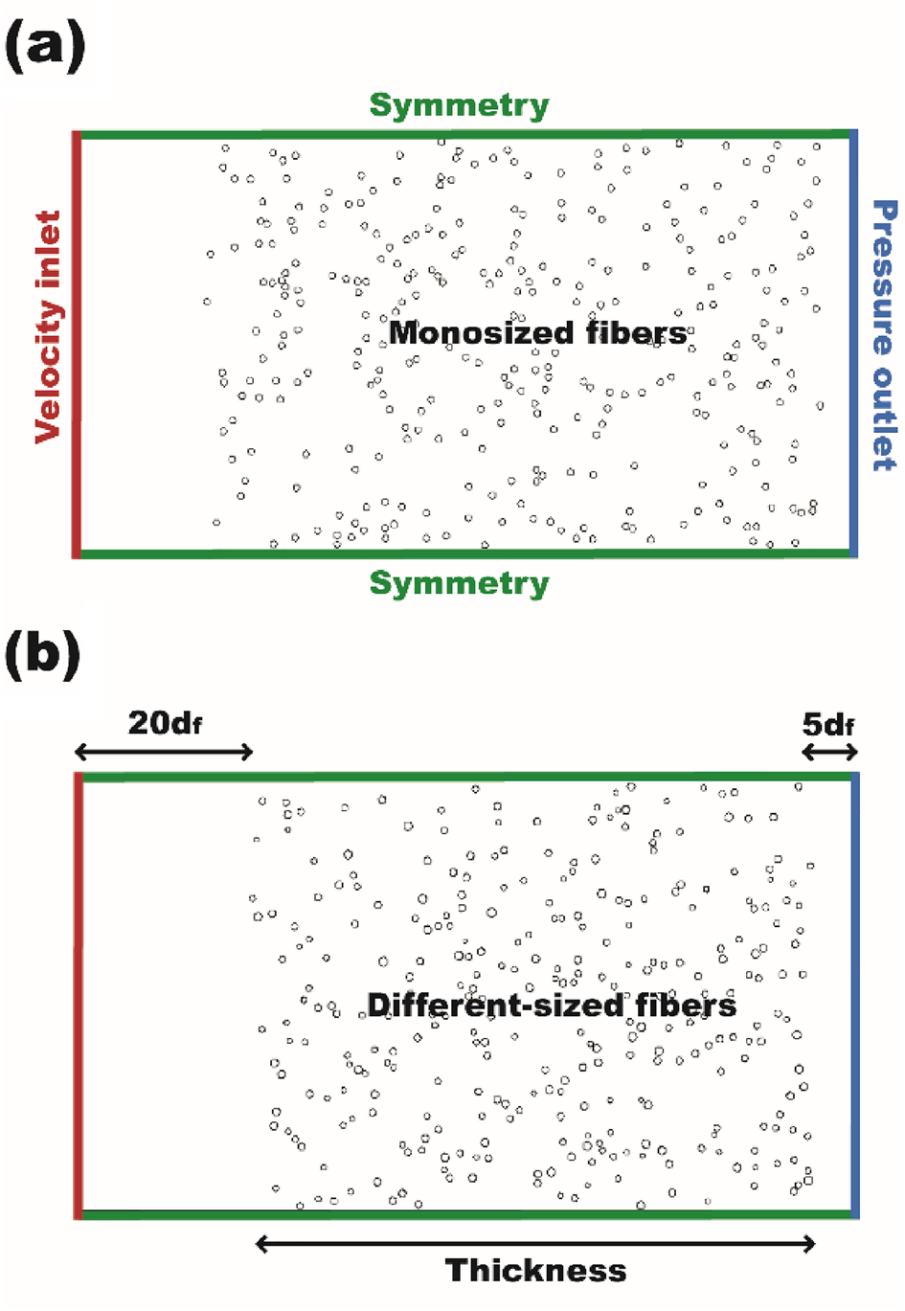
Calculation domains with boundary conditions for (**a**) a monosized fibrous filter and (**b**) a different-sized fibrous filter.

In this study, a MATLAB code was developed to generate 2-D fibrous structures in the calculation domain. Using the fiber generating algorithm, circular fibers of a specific diameter were randomly placed in a pre-defined area sequentially until the designated packing density was achieved. During the generation of each fiber, the distances between a newly created fiber and existing fibers were continuously monitored to avoid a fiber overlap. Moreover, to ensure a high quality of meshes, the minimum distance between the adjacent fiber centers was set to 1.1*d*_*f*_. It should be noted that the monosized fibers were applied for the numerical cases as presented in Table 1 to develop the relationship between $\frac{{C_{D} }}{2} \cdot {\text{Re}}$, other filtration parameters, and pressure drop, but the fitted log-normal fiber size distributions, namely, different-sized fibers, were adapted in the validation of the numerical methods by simulating the fabricated electrospun nanofibers, which were examined using a scanning electron microscope (SEM). The exemplary calculation domains with the monosized and different-sized fibrous geometries are shown in Fig. 1a,b, respectively. Each calculation domain has more than 300 fibers. This was achieved by adjusting the height of the domain to ensure that simulation results are independent of the statistical uncertainty^41^.

The generated geometries were imported in Gambit software to construct meshes and set boundary conditions. Approximately more than 1,000,000 triangular mesh elements were used with the high mesh density around each fiber for the rapid changes in the dependent variables, e.g., pressure and velocity. The mesh independence study was performed by increasing the number of grid points around a fiber. We observed that the obtained pressure drops across the filter media for the number of grid points ranging from 30 to 80 remained unchanged. Therefore, we selected more than 40 grid points to ensure most efficient use of computing power. As shown in Fig. 1, the inlet and outlet are placed at a distance of 20*d*_*f*_ upstream and 5*d*_*f*_ downstream from the front and rear ends of the filters, respectively, and the velocity inlet and pressure outlet boundary conditions were applied in these cases, respectively. The symmetric boundary condition was set to both upper and lower sides of the calculation domain.

#### Flow field calculation

After generating the calculation domains, they were exported to ANSYS Fluent v18.0 software to solve the governing equations, i.e., continuity, momentum, and energy equations. Owing to considerably low Re based on the fiber diameter less than unity, the flow through the fibrous filter is dominated by viscosity and is unaffected by gravity and inertia. Therefore, the flow characteristic can be well described by the Stokes flow. In the numerical analysis, the solved continuity and momentum equations are:

4 $$ \nabla \cdot U = 0 $$

and

5 $$ \nabla P - \mu \nabla^{2} U = 0. $$

**Figure 2:**
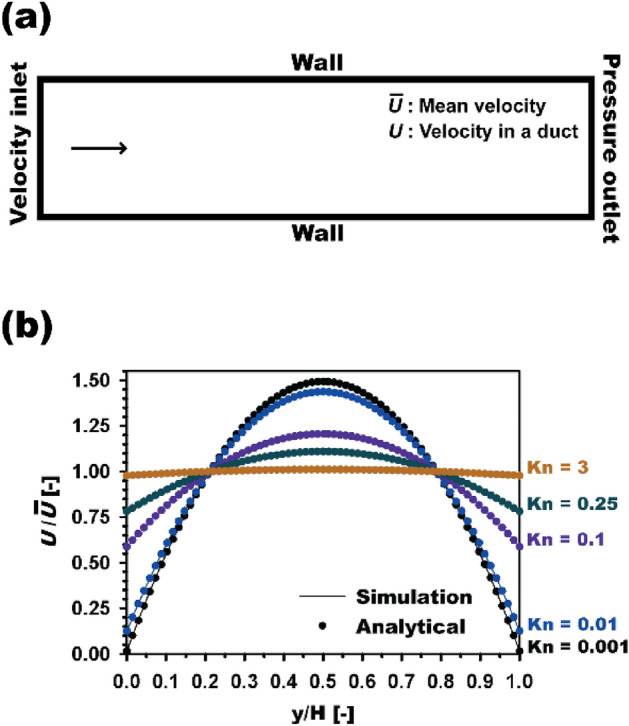
(**a**) Calculation domain with boundary conditions for a 2-D duct flow and (**b**) comparison of velocity profiles at different Kn ranging from 0.001 to 3.

It is well-known that aerodynamic slip of a gas flow occurs when the gas flows around nanofibers, which have comparable sizes to the mean free path of gas molecules. A significant aerodynamic slip occurs in the slip-flow regime (0.001 < Kn < 0.25) and transition regime (0.25 < Kn < 10). This observation is applicable to typical electrospun nanofibers. The slip effect was applied by enabling the low-pressure slip boundary function in the Fluent software, which is based on the first-order Maxwell model. Using this model, the slip velocity (*U*_*w*_) was applied as a boundary condition to the fibrous media as follows:

6 $$ U_{w} = \left( {\frac{{2 - \sigma_{\nu } }}{{\sigma_{\nu } }}} \right)\lambda \frac{\partial U}{{\partial n}}, $$

where, *σ*_*v*_ is the momentum accommodation coefficient. It should be noted that the slip boundary condition on external flows, e.g., flow over cylinders or fibers, has not yet been thoroughly investigated using the analytical expressions. Therefore, to verify the application of this boundary condition to the simulations, the Poiseuille flow in a 2-dimensional duct was examined. Moreover, the flow velocity profiles at the outlet were compared to the well-established analytical solution of the fully developed slip velocity, which can be expressed as follows^42^:

7 $$ U\;({\text{y}}) = 6\overline{U}\left( {\frac{{\text{y}}}{{\text{H}}} - \frac{{{\text{y}}^{2} }}{{{\text{H}}^{2} }} + 2\left( {\frac{{2 - \sigma_{\nu } }}{{\sigma_{\nu } }}} \right){\text{Kn}}} \right)/\left( {1 + 12\left( {\frac{{2 - \sigma_{\nu } }}{{\sigma_{\nu } }}} \right){\text{Kn}}} \right), $$

where, $\overline{U}$ is the mean velocity, *σ*_*v*_ is 0.9137 in this case^14,43^. The tested calculation domain is shown in Fig. 2a. The duct length is sufficiently long to achieve a fully developed flow. We considered duct heights of 3.35 × 10^−5^, 3.35 × 10^−6^, 3.35 × 10^−7^, 1.34 × 10^−7^ and 1.12 × 10^−8^ m, which correspond to the gap-based Kn ($= \frac{\lambda }{2H}$) of 0.001, 0.01, 0.1, 0.25 and 3, respectively, when the mean free path of air molecules is 67 nm. Figure 2b shows the slip velocity profiles obtained numerically (line) and analytically (symbol). The results show that velocities at the walls increase with as Kn increases. The numerical velocity profiles for all Kn cases match accurately with the analytical results.

### Experiment

Further verification of simulation results was performed using electrospun nanofibers fabricated in the lab to compare the pressure drops obtained by numerical analysis and experiments. For the fabrication of electrospun nanofibers, polyacrylonitrile (PAN) polymer (Sigma-Aldrich, Co. LLC., USA) was dissolved in N,N-dimethylformamide solution (Sigma-Aldrich, Co. LLC., USA) with predetermined concentrations ranging from 7 to 11 wt% to obtain different fiber sizes. The polymer solutions were gently stirred for 12 h at 50 °C with a magnetic stirrer to obtain a homogeneous PAN solution.

**Figure 3:**
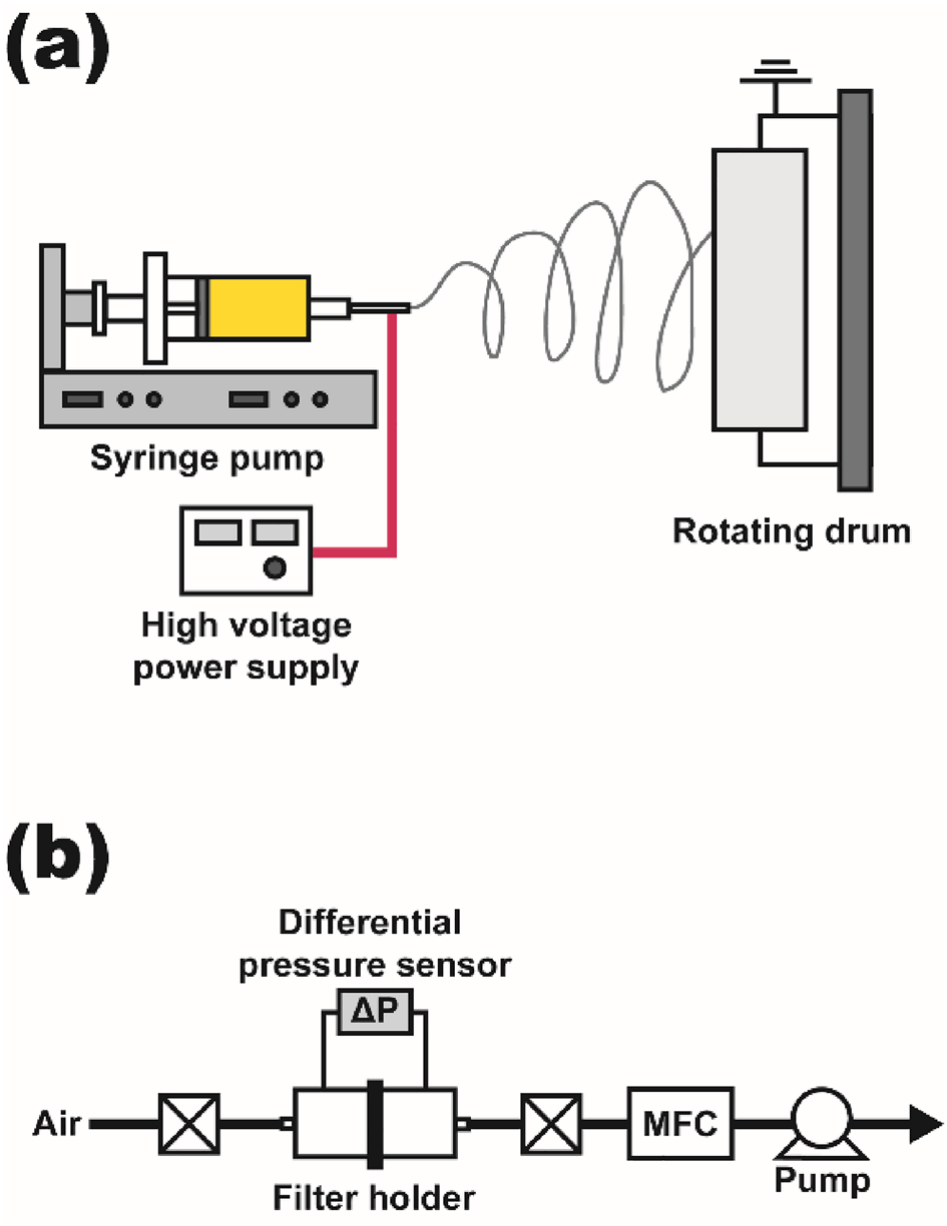
Schematics of the experimental setup for (**a**) fabrication of electrospun nanofibers and (**b**) pressure drop measurement.

Figure 3a represents the electrospinning setup with a rotating drum system. The 12-ml syringe was filled with a PAN solution and was installed in a syringe pump. A negative high voltage supply was connected to the needle (19–23-gauge), and the drum was grounded as a collector. The electrospun nanofibers were collected on a non-woven substrate sheet covered around the drum. The solution feed rate (0.3–0.5 ml h^−1^) and high voltage (10–20 kV) were carefully adjusted to achieve a stable generation of nanofibers without any event of solution dripping or intermittence during the entire electrospinning process. The information on the fabricated electrospun nanofiber filters is presented in Table S1 in the Supplementary material. The ranges of mean fiber size, packing density, and thickness of the filters vary from 280 to 900 nm, 2.4 to 7.4%, and 9 to 120 µm, respectively. After the electrospinning process, collected nanofiber samples were carefully collected from the drum and were dried in an oven at 50 °C for 2 h for complete solvent evaporation before the pressure drop measurements were conducted. Figure 3b shows the schematic of the experimental setup used for pressure drop measurement. We used a filter holder with an inner diameter of 36 mm, and high- and low-pressure ports of the differential pressure sensor (model 985 M, Beck Sensortechnik GmbH, Germany) were connected before and after the filter, respectively. Clean air was supplied by using a vacuum pump, and the flow rate through the filter was adjusted by a mass flow controller to set the face velocity to 5.3 cm/s.

**Figure 4:**
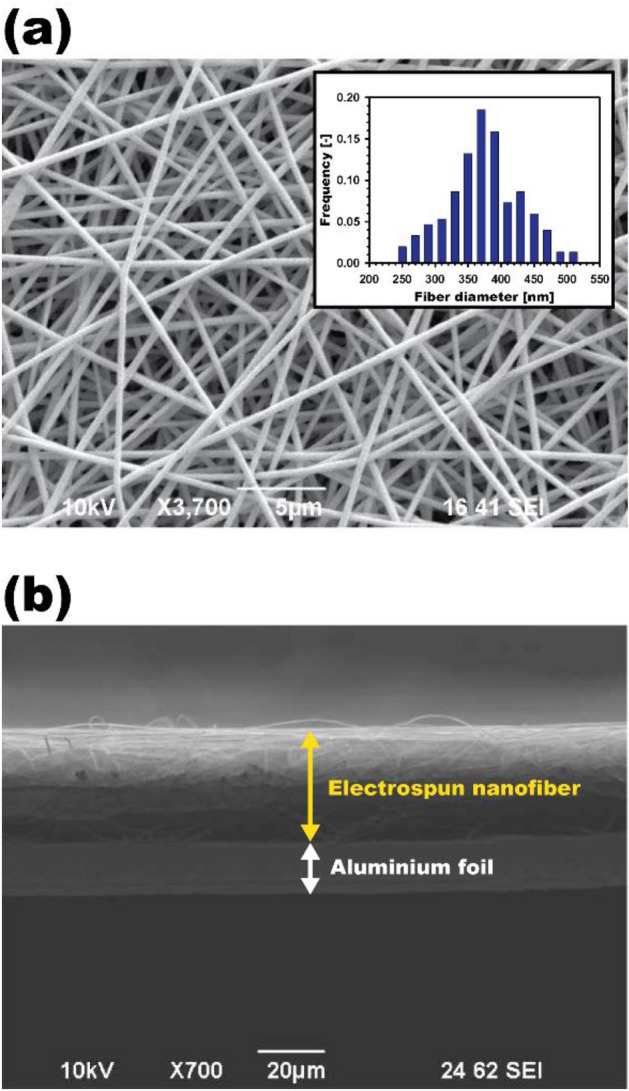
SEM images of 8 wt% PAN electrospun nanofiber: (**a**) front view for the fiber size measurement (embedded figure: fiber size distribution of nanofibers); (**b**) side view for the thickness measurement.

Fiber diameter and thickness of electrospun nanofiber filters collected on an aluminum foil were characterized using an SEM. Figure 4 shows the exemplary SEM images for the diameter and thickness measurements, which were obtained using the image-processing software ImageJ. Note that some researchers used a thickness gauge for the measurement of filter thickness, but electrospun nanofiber layers are extremely thin and vulnerable to this measurement process, which might underestimate the thickness. We confirmed that the SEM analysis can be used to precisely obtain the thickness without any measurement distortion.

The packing density (*α*) of electrospun nanofiber filters, i.e., the volume fraction of fibers in a filter, can be calculated as follows:

8 $$ \alpha = \frac{{V_{fiber} }}{{V_{filter} }} = \frac{{m_{fiber} }}{{\rho_{fiber} \times L \times A}}, $$

where, *V*_*fiber*_ and *V*_*filter*_ are the volumes of fiber and filter (47-mm circular filter coupon in this study), respectively. *m*_*fiber*_ is the fiber mass, which is measured using a microbalance. *L* is the thickness of the fiber layer, examined by the SEM analysis as shown in Fig. 4b. *A* is the frontal area of a 47-mm circular filter coupon and is 1735 mm^2^.

## Results and discussion

### Verification of numerical pressure drops

**Figure 5:**
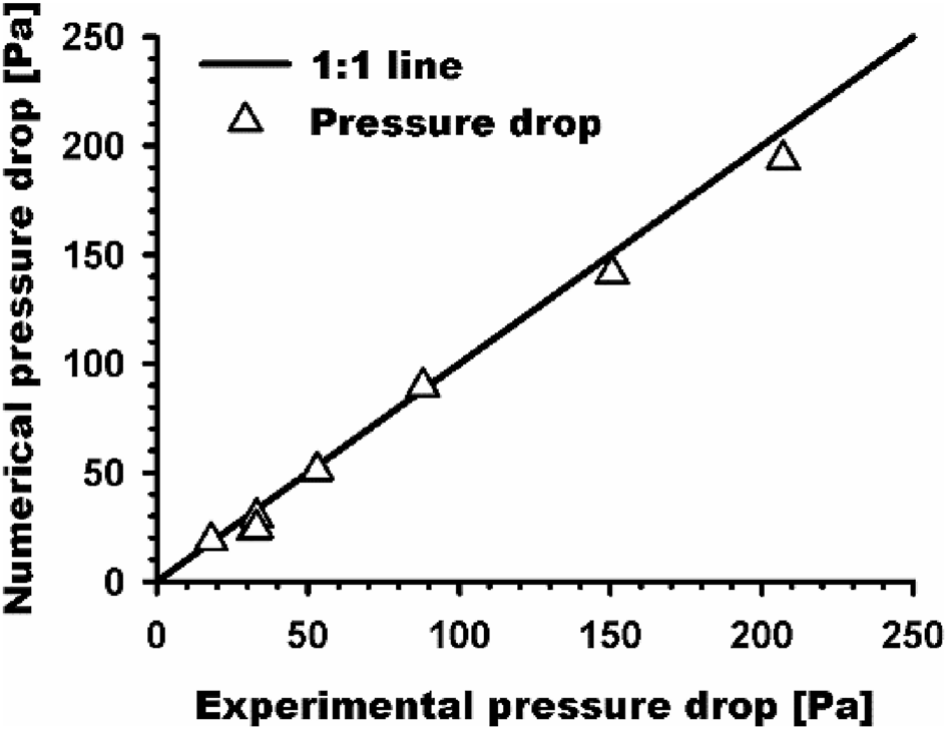
Comparison of the measured and simulated pressure drops for electrospun nanofiber filters.

The pressure drops for eight cases obtained by numerical simulations and experiments are shown in Fig. 5 for the verification of the accuracy of simulation methods. The measured pressure drops ranged from approximately 18–200 Pa. The line represents the 1:1 line between the experimental and numerical results. From comparison of results, we observed that the obtained experimental and numerical pressure drops for the tested cases with various fiber sizes and thicknesses agreed well for each case, and the calculated average relative error was less than 10%.

### Effect of flow velocity

**Figure 6:**
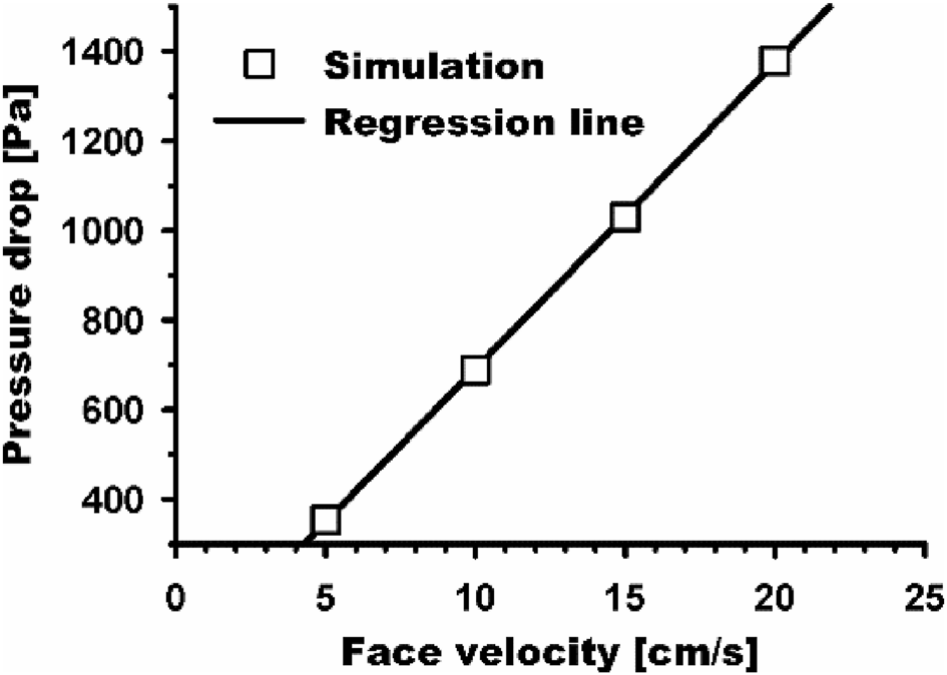
Relationship between face velocity and pressure drop on nanofiber filters. The line represents a fitting curve with a square root value higher than 0.99.

Figure 6 plots the relationship between the face velocity and pressure drop. Pressure drops at four different velocities of 5, 10, 15, and 20 cm/s were examined for the filters with the 200 nm fibers and the 40 µm thickness. As shown in Eq. (3), the pressure drop has a linear relationship with the velocity, which can be clearly seen in the simulation results. The linear relationship also indicates that $\frac{{C_{D} }}{2} \cdot {\text{Re}}$ is not a function of flow velocity in the case of nanofiber filters.

### Effect of thickness

**Figure 7:**
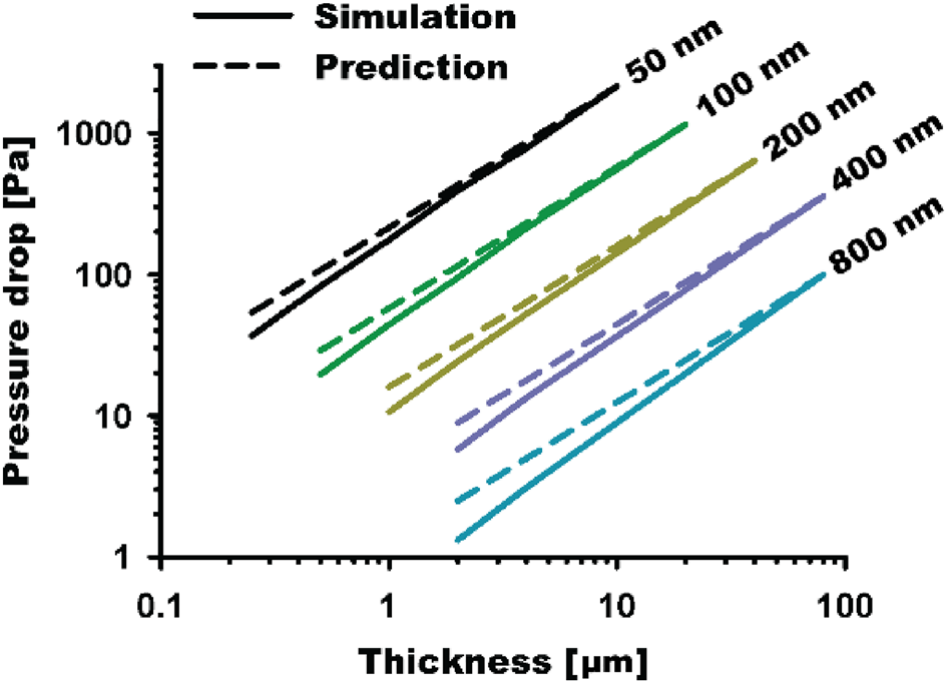
Relationship between filter thickness and pressure drop on nanofiber filters with the fiber sizes of 50, 100, 200, 400, and 800 nm.

The effect of thickness of nanofiber filters is intensively examined based on fiber size. Figure 7 shows the relationship between the thickness and pressure drop on the fiber sizes of 50, 100, 200, 400, and 800 nm at the packing density of 0.06. Table 2 provides the numerically obtained pressure drop values. In Fig. 7, the numerical pressure drops are denoted as solid lines, and the dashed lines represent the theoretical linear values of (*L/L*_*max*_)·Δ*P*_*max*_, assuming that the pressure drops decrease linearly, starting from the maximum pressure drop values, with the thickness ratio. Additionally, the slight deviations from the perfect linear lines, marked as dashed lines, can be observed in Fig. 7. These deviations become significant for the thinner layers. Based on the conventional theory, the pressure drop through a filter is proportional to the filter thickness. For example, a pressure drop of 1151 Pa is observed for the conditions of *d*_*f*_ = 100 nm, *α* = 0.06, *U*_*0*_ = 5 cm/s, and *L* = 20 µm. Therefore, the theoretically estimated pressure drop of the 0.5-µm thickness filter under the same conditions should be 28.8 Pa (= 0.5/20 × 1151 Pa), which gives approximately 32% relative difference from the numerical pressure drop value of 19.5 Pa. We expect that these deviations result from the fiber arrangement, especially in the thin nanofiber layers. When the layer of nanofibers is thin, only few fiber elements are composed of the layer, e.g., cases of *d*_*f*_ = 50 and 100 nm and *L* = 0.25 and 0.5 µm, respectively. Notably, for electrospun nanofibers are generally fabricated as a significantly thin layer, this non-linearity between the thickness and pressure drop should be considered during the pressure drop prediction for electrospun nanofibers.

**Table 2:** Filter thickness and pressure drop values for the different cases plotted in Fig. 7.

Fiber diameter *d*_*f*_ (nm)	Filter thickness *L* (µm)	Numerical pressure drop Δ*P* (Pa)	Predicted pressure drop (*L*/*L*_*max*_)·Δ*P*_*max*_ (Pa)	Relative difference (%)
50	0.25	36.7	53.6	31.5
	0.5	82.4	107.3	23.2
	1	175.8	214.5	18.0
	2	391.6	429.0	8.7
	4	779.1	858.1	9.2
	10 (*L*_*max*_)	2145.2 (Δ*P*_*max*_)	2145.2	–
100	0.5	19.5	28.8	32.3
	1	44.2	57.6	23.3
	2	94.8	115.2	17.7
	4	212.2	230.4	7.9
	20 (*L*_*max*_)	1151.1 (Δ*P*_*max*_)	1151.1	–
200	1	10.6	15.9	33.3
	2	24.2	31.9	24.1
	4	52.3	63.8	18.0
	40 (*L*_*max*_)	637.6 (Δ*P*_*max*_)	637.6	–
400	2	5.8	8.9	34.8
	4	13.4	17.8	24.7
	80 (*L*_*max*_)	355.5 (Δ*P*_*max*_)	355.5	–
800	2	1.3	2.5	48.0
	4	3.1	5.0	38.0
	80 (*L*_*max*_)	99.5 (Δ*P*_*max*_)	99.5	–

### Effects of packing density and Knudsen number

**Figure 8:**
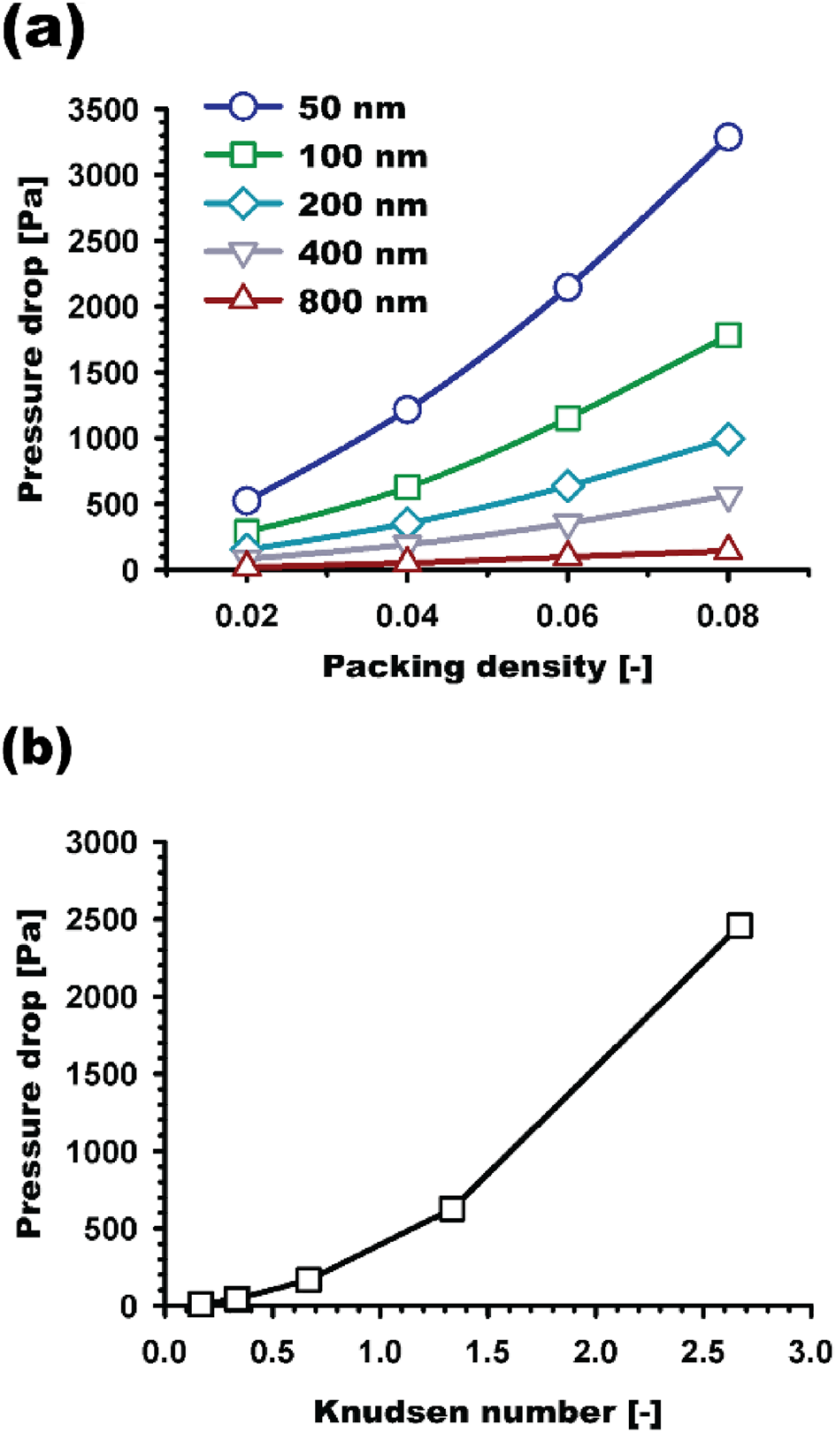
Pressure drop values obtained for the nanofiber filters according to (**a**) packing density and (**b**) Kn.

Figure 8a shows the pressure drops obtained for each fiber size, i.e., 50, 100, 200, 400, and 800 nm, according to the packing densities ranging from 0.02 to 0.08; all other conditions were the same for all data points in each curve. Moreover, based on fiber size, the effect of Kn, ranging from 0.17 to 2.67, was examined, and the results are plotted in Fig. 8b. We found that the packing density and Kn have a non-linear relationship with the pressure drop.

When doubling the packing density, e.g., from 0.02 to 0.04 and from 0.04 to 0.08, the pressure drops are estimated to be more than doubled and reach an approximately 2.2–2.9 times higher value for the higher packing density. The increased packing density results in increasing not only the surface area of the fibers but also the flow velocity inside the filter, which creates an additional pressure drop through the filter media.

### Correlation equation for predicting pressure drops

Based on Eq. (2), the product of drag coefficient and Re, i.e., $\frac{{C_{D} }}{2} \cdot {\text{Re}}$, can be represented as follows:

9 $$ \frac{{C_{D} }}{2} \cdot {\text{Re}} = \frac{\pi }{4} \cdot \frac{{d_{f}^{2} }}{{\mu \alpha U_{0} L}} \cdot \Delta P. $$

The numerically obtained pressure drops and other parameters for all cases were inserted into Eq. (9) to get $\frac{{C_{D} }}{2} \cdot {\text{Re}}$. Moreover, in Eq. (3), we assumed that the obtained values for $\frac{{C_{D} }}{2} \cdot {\text{Re}}$ are a function of packing density, Kn, and the ratio of thickness to fiber diameter and that they can be expressed in terms of power functions of these properties as follows:

10 $$ \frac{{C_{D} }}{2} \cdot {\text{Re}} = f\left( {\alpha ,\;{\text{Kn}},\;\frac{L}{{d_{f} }}} \right) = C_{1} \cdot \alpha^{{C_{2} }} \cdot {\text{Kn}}^{{C_{3} }} \cdot \left( {\frac{L}{{d_{f} }}} \right)^{{C_{4} }} . $$

We used an ordinary least-squares regression method to obtain *C*_*1*_, *C*_*2*_, *C*_*3*_, and *C*_*4*_ and found the best fitting curves with two thickness ranges for the simulation conditions examined in this study as follows:

11 $$ \frac{{C_{D} }}{2} \cdot {\text{Re}} = 14.5263\alpha^{0.3821} \cdot {\text{Kn}}^{ - 0.1262} \cdot \left( {\frac{L}{{d_{f} }}} \right)^{0.1128} . $$

Moreover, the correlation equation for predicting the pressure drops on nanofiber filters can be obtained by inserting Eq. (11) into Eq. (9):

12 $$ \Delta P = 18.4955\frac{{\mu \alpha^{1.3821} U_{0} }}{{d_{f} }}{\text{Kn}}^{ - 0.1262} \left( {\frac{L}{{d_{f} }}} \right)^{1.1128} . $$

**Figure 9:**
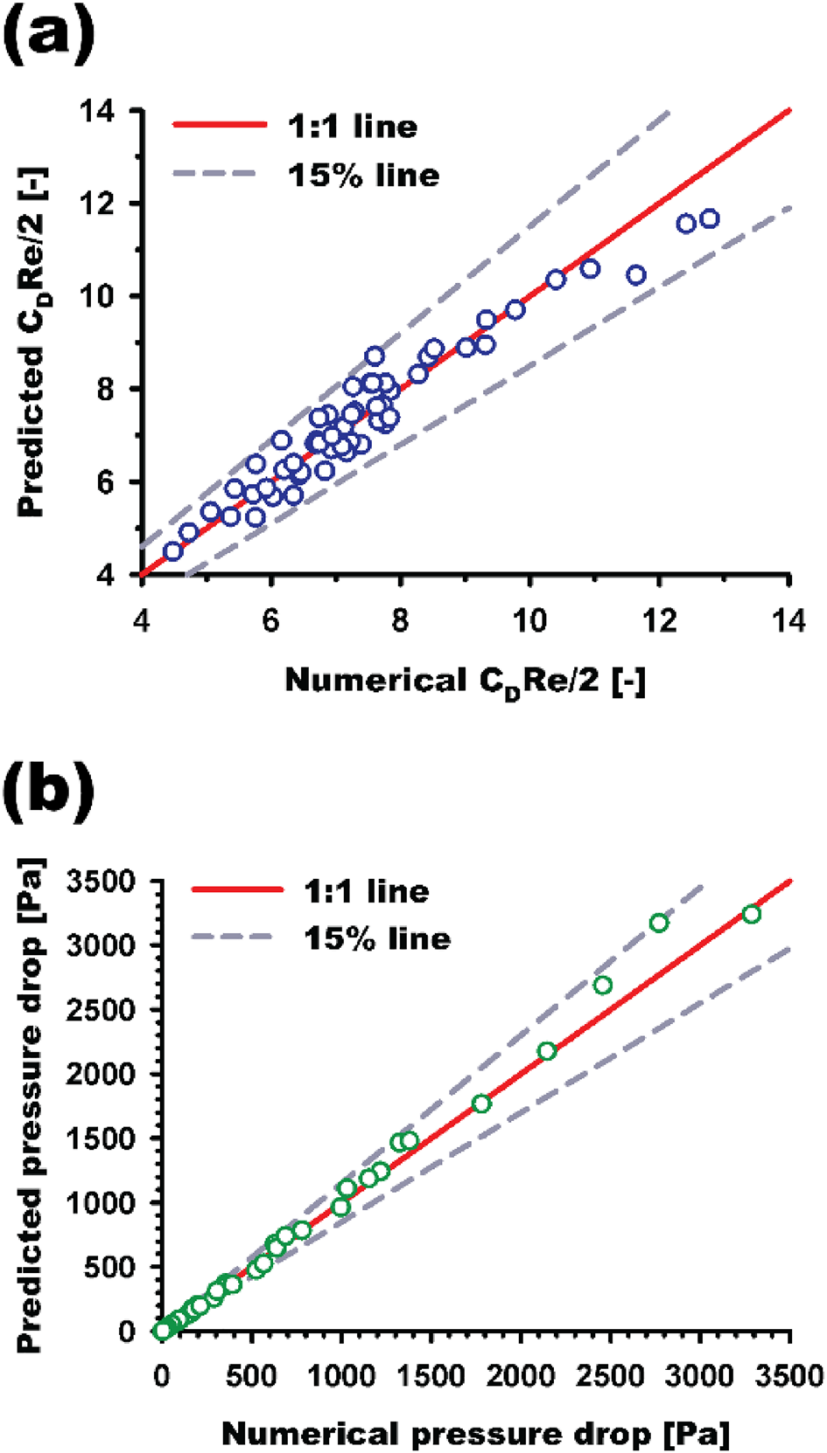
Comparison between the numerical and predicted values of (**a**) $\frac{{C_{D} }}{2} \cdot {\text{Re}}$ and (**b**) pressure drop for all simulation cases.

The comparisons of the numerical results and predicted values of $\frac{{C_{D} }}{2} \cdot {\text{Re}}$ (Eqs. (9) and (11)) and pressure drop are shown in Figs. 9a,b, respectively. The results highly agree and have relative errors of less than 15% in all cases, and the average relative errors of 56 cases for both $\frac{{C_{D} }}{2} \cdot {\text{Re}}$ and pressure drop are less than 1%.

**Figure 10:**
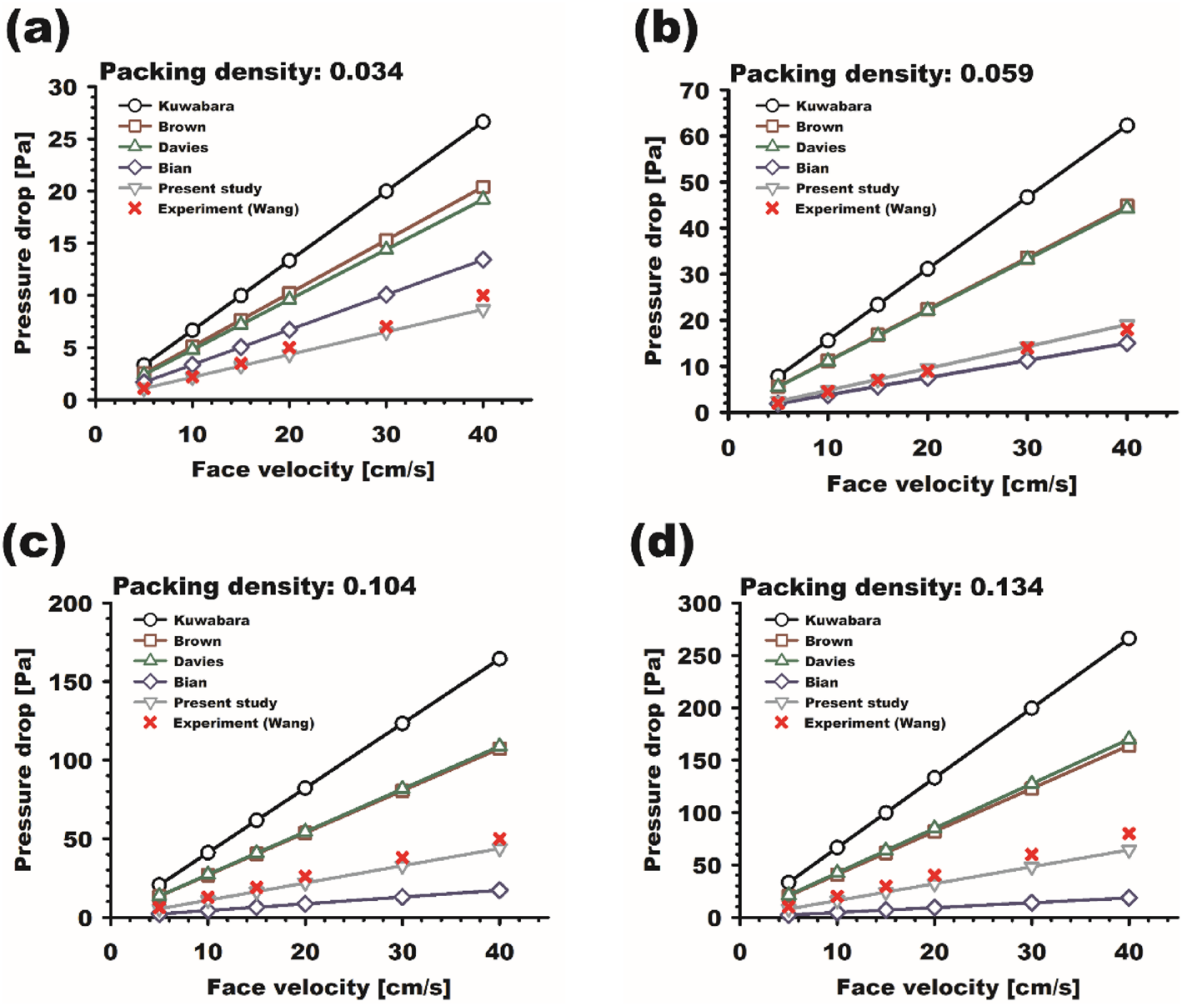
Comparison between pressure drops obtained by theoretical approaches, empirical correlations, present model, and experiments for ultra-thin single-layer nanofiber filters with different packing densities of (**a**) 0.034, (**b**) 0.059, (**c**) 0.104, and (**d**) 0.134 as a function of face velocity.

Finally, we compared pressure drops estimated by our developed model with those from previous works. Figure 10 shows a comparison of pressure drops across ultra-thin nanofiber filters with a thickness and fiber diameter of 150 nm (i.e., single-layer nanofibers) as a function of face velocity. The experimental data for filters with different packing densities of 0.034, 0.059, 0.104, and 0.134 were obtained from Wang et al.^18^. The theoretical models from Kuwabara^34^ and Brown^36^ and empirical correlations from Davies^35^ and Bian et al.^37^ are described in the Supplementary material. The results in Fig. 10 indicate that, in general, pressure drops estimated by models from Kuwabara, Brown, and Davies tend to be overestimated compared to experimental pressure drops across the ultra-thin nanofiber filters. Furthermore, the empirical model of Bian et al. shows good agreement only for the case of 0.059 packing density, while for other cases, inconsistencies may arise due to potential issues that can arise from using an empirical approach. However, our developed model (Eq. 12) predicts pressure drops that show good agreement with the experimental data for all packing densities, as shown in Fig. 10. Based on these results, it can be concluded that slip effects and thickness should be carefully considered together in the prediction of pressure drop for extremely thin nanofiber filters.

## Conclusion

In this study, we obtained the correlation equation for the prediction of pressure drop across electrospun nanofiber filters. This equation was developed from the computational fluid dynamics simulations of 56 cases of these nanofiber filters. In the simulations, the effects of packing density, thickness, face velocity, and fiber size on the pressure drop were examined, and the relationship between these parameters and drag-term (i.e., $\frac{{C_{D} }}{2} \cdot {\text{Re}}$) was studied. We assumed that, for electrospun nanofiber filters, due to their nano-sized fibers and extremely thin layers, the product of drag coefficient and Re is a function of packing density, Kn, and the ratio of thickness to fiber diameter; this is different from the drag theory on conventional filters. Finally, the correlation equation for predicting pressure drops across nanofiber filters with randomly distributed fibers were derived. We also provided pressure drops estimated by the correlation equation for the wide range of face velocities from 5 to 20 cm/s in Fig. S1 in the Supplementary material for readers who need to refer to the values. To ensure realistic values for filtration applications, only pressure drops up to 1000 Pa are presented. Based on the data in Fig. S1, readers can refer to the pressure drops according to different fiber sizes (50, 100, 200, 400, and 800 nm), thicknesses (1, 2, 5, 10, and 20 µm), and packing densities (0.02, 0.04, 0.06, and 0.08) at the velocity range.

The prediction model developed in this study has some limitations. First, the developed correlation equation might be inapplicable to conventional fibrous filters, which have a relatively broader size distribution of fibers because the model was developed considering the mono-sized fibers. Therefore, electrospun nanofiber filters were considered as good candidates because they generally consist of a narrow size distribution of fibrous media. Therefore, in the simulations, the velocity range was limited to the range of 5–20 cm/s to target the usage of electrospun nanofiber filters, which are applicable in window screening for natural ventilation and face masks. Second, the simulations were performed for clean filters and provides the initial filter pressure drops without dust loading. Because the loading effect is much more complex and is significantly altered by various filtration conditions that can affect dendrite formation of particles, we expect that the intensive experimental approach might be more effective than numerical models. Despite all these limitations, the developed simulation methods and derived equation for predicting pressure drops on air filtration system are applicable in various applications. The developed model in this study is not limited to nanofibers produced only by the electrospinning process. There are various methods for creating nanofibers, such as solution blowing and centrifugal spinning methods^44–48^. If the experimental conditions used in this study are met or if the nanofibers have a narrow fiber size distribution, the model developed in this study can be applied to predict pressure drop for nanofibers produced by these methods as well.

## Supplementary Information

Supplementary Information.

## Data Availability

Some or all data and models that support the findings of this study are available from the corresponding author upon reasonable request.
