# Supplementary material for: Modeling pressure drop values across ultra-thin nanofiber filters with various ranges of filtration parameters under an aerodynamic slip effect
Source: Sci Rep. 2023 Apr 3;13:5449. doi: 10.1038/s41598-023-32765-4 (PMC10070425; doi:10.1038/s41598-023-32765-4)
Supplement: Supplementary file 1 — Supplementary Information. [file 41598_2023_32765_MOESM1_ESM.docx]

***Supplementary material***

**Modeling pressure drop values across ultra-thin nanofiber filters with various ranges of filtration parameters under an aerodynamic slip effect**

Songhui Lee^1^, Dai Bui-Vinh^1^, Minwoo Baek^1^, Dong-Bin Kwak^2,*^, Handol Lee^1,*^

*^1^Department of Environmental Engineering, Inha University, 100 Inha-ro, Michuhol-gu, Incheon, 22212, Republic of Korea*

*^2^Particle Technology Laboratory, Mechanical Engineering, University of Minnesota, 111 Church St., S.E., Minneapolis 55455, USA*

*Corresponding authors

*Email address: kwak0068@umn.edu (Dong-Bin Kwak)*

*Tel.: +1 612 626 1517; Fax: +1 612 625 6069*

*Email address: leehd@inha.ac.kr (Handol Lee)*

*Tel.: +82 32 860 7504; Fax: +82 32 865 8625*

**Table S1.** Information on fabricated nanofiber filters.

| **Case** | **Mean fiber diameter [µm]** | **Standard deviation [µm]** | **Packing density [%]** | **Thickness [µm]** |
| --- | --- | --- | --- | --- |
| **1** | **0.280** | **0.054** | **2.79** | **9.3** |
| **2** | **0.373** | **0.054** | **4.23** | **33.2** |
| **3** | **0.446** | **0.058** | **3.68** | **110.2** |
| **4** | **0.510** | **0.048** | **3.62** | **14.4** |
| **5** | **0.534** | **0.089** | **5.47** | **72.2** |
| **6** | **0.616** | **0.083** | **7.40** | **19.1** |
| **7** | **0.779** | **0.110** | **4.53** | **29.8** |
| **8** | **0.858** | **0.171** | **2.46** | **105.8** |

Eight cases of electrospun nanofiber filters were examined for the validation of the accuracy of the numerical simulations. For the reliable measurements of the nanofiber weights for the calculation of accurate packing density, we collected nanofibers on the substrate with the sufficient electrospinning time of 2 to 10 h. Moreover, we focused on the stable electrospinning process to get the constant fiber size during the fabrications by choosing the proper high voltage, solution feed rate, and distance between the needle tip and collecting drum.


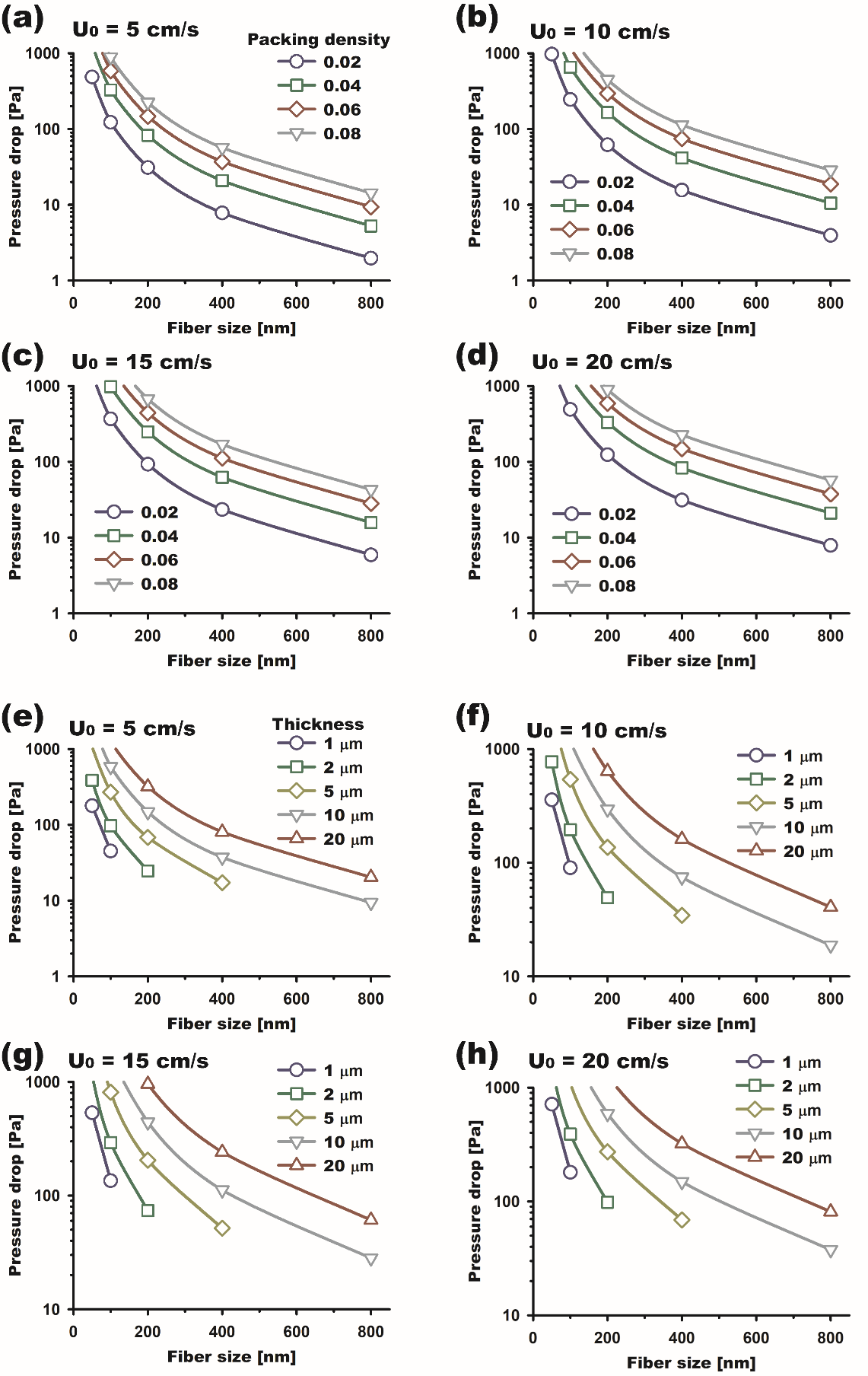


**Figure S1.** The pressure drops estimated by the model in this study: (a-d) different packing densities at *U_0_* = 5, 10, 15, 20 cm/s and *L* = 10 µm; (e-h) different thicknesses at *U_0_* = 5, 10, 15, 20 cm/s and *α* = 0.06.

***Prediction of pressure drops across fibrous filters***

1. Kuwabara model^1^

$\Delta P_{Kuwabara}=\frac{U_{o}\mu L}{d_{f}^{2}}\times\frac{16\alpha}{\mathrm{Ku}}$. (S1)

with

$\mathrm{Ku}=-0.5\ln\alpha-0.75+\alpha-0.25\alpha^{2}$. (S2)

2. Brown model^2^

$\Delta P_{Brown}=\frac{U_{o}\mu L}{d_{f}^{2}}\times\frac{16\alpha\left( 1+1.996\mathrm{Kn} \right)}{\mathrm{Ku}+1.996\mathrm{Kn}(\mathrm{Ku}+0.5\left( 1-\alpha\right)^{2})}$ (S3)

3. Davies model^3^

$\Delta P_{Davies}=\frac{U_{o}\mu L}{d_{f}^{2}}\times64\alpha^{1.5}\left( 1+56\alpha^{3} \right)$ (S4)

4. Bian et al. model^4^

$\Delta P_{Bian}=\frac{1.364}{\pi}\frac{\mu U_{o}L}{d_{f}^{2}}\frac{\alpha^{0.158}}{1-\alpha} Kn^{-0.556}$. (S5)

where *U_0_* is the face velocity, *μ* is the fluid dynamic viscosity, *L* is the filter thickness, *α* is the fiber packing density, *d_f_* is the fiber diameter, Kn is the Knudsen number ($Kn=\frac{2\lambda}{d_{f}}$).

Reference

1. Kuwabara, S. The forces experienced by randomly distributed parallel circular cylinders or spheres in a viscous flow at small Reynolds numbers. *Journal of the Physical Society of Japan* vol. 14 527–532 (1959).
2. Brown, R. C. *Air Filtration: An Integrated Approach to the Theory and Applications of Fibrous Filters*. (Pergamon Press, 1993).
3. Davies, C. N. The separation of airborne dust and particles. *Proc. Inst. Mech. Eng.* **167**, 185–213 (1953).
4. Bian, Y., Zhang, L. & Chen, C. Experimental and modeling study of pressure drop across electrospun nanofiber air filters. *Build. Environ.* **142**, 244–251 (2018).
